# Fitness gains hamper efforts to tackle drug resistance

**DOI:** 10.7554/eLife.01809

**Published:** 2013-12-10

**Authors:** Shanta Dutta

**Affiliations:** 1 **Shanta Dutta** is in the Bacteriology Division, National Institute of Cholera and Enteric Diseases, Kolkata, Indiashanta1232001@yahoo.co.in

**Keywords:** *Salmonella*, typhoid, fitness cost, epistasis, fluoroquinolone, antibiotic resistance, *Salmonella enterica serovar* Typhi

## Abstract

It has long been assumed that resistance to antibiotics reduces the fitness of disease-causing bacteria, but experiments on *Salmonella* Typhi, the bacteria that causes Typhoid fever, are now challenging this view.

**Related research article** Baker S, Duy PT, Nga TVT, Dung TTN, Voong VP, Chau TT, Turner AK, Farrar J, Boni MF. 2013. Fitness benefits in fluoroquinolone-resistant *Salmonella* Typhi in the absence of antimicrobial pressure. *eLife* **2**:e01229. doi: 10.7554/eLife.01229

**Image** DNA pyrosequencing (y-axis) and colony counting (x-axis) provide similar estimates for the frequency of antibiotic resistance mutations

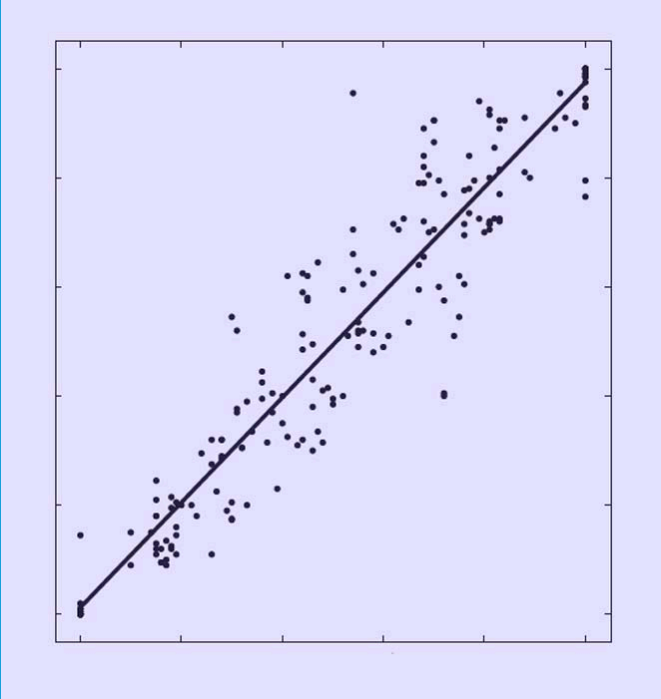

The effectiveness of the antibiotics used to treat diseases caused by bacteria is increasingly being limited by the spread of resistance to these drugs. For a long time it was thought that most of the mutations that lead to antimicrobial resistance in bacteria led to a reduction in fitness, which meant that the resistant bacteria did not grow as fast as those that were susceptible to antimicrobials. These fitness costs are important because they determine how quickly resistance is likely to develop, or to disappear if antibiotic use is discontinued (Andersson and Hughes, 2010). However, resistant bacteria often develop secondary mutations that can compensate for these fitness costs, and these mutations allow the bacteria to thrive, even in antimicrobial free environments (Lenski, 1998). When this occurs, resistance is likely to continue even if antibiotic use is stopped. This is why public health bodies around the world are so worried by the spread of antibiotic resistance.

Typhoid fever is caused by eating food or drinking water contaminated with *Salmonella* Typhi bacteria. According to the World Heath Organisation (WHO), the drug of choice for treating typhoid fever is ciprofloxacin, which belongs to the fluoroquinolone family of antibiotics (WHO, 2003). However, resistance to the fluoroquinolones is increasing all over the world (Parry and Beeching, 2009). Now, in *eLife*, Stephen Baker of the Oxford University Clinical Research Unit in Vietnam and co-workers in Vietnam and the UK have compared the fitness of various mutants of *Salmonella* Typhi that are resistant to fluoroquinolones with the fitness of a strain that is still susceptible to these drugs (Baker et al., 2013).

The fluoroquinolones target enzymes that untangle knots in bacterial genomes. Resistance to these antibiotics in *Salmonella* Typhi involves changes in these target enzymes that reduce the ability of the drugs to bind to them. These changes occur by substitution mutations in two genes, *gyr*A and/or *par*C, that encode these enzymes. Advances in site-directed mutagenesis allowed Baker and co-workers to construct eleven fluoroquinolone-resistant mutants and one susceptible control mutant with known nucleotide substitutions from a susceptible, weakened laboratory strain of *Salmonella* Typhi. The resistant mutants were generated by substitutions at two positions in the *gyr*A gene and one in the *par*C gene, which were introduced in combinations to create single, double or triple mutants. Seven of the eleven lab-engineered mutants have been found in clinical isolates, whilst the remaining four had not been observed in nature before.

To start each in vitro competition assay, a series of cultures containing equal numbers of bacteria from the susceptible parent strain and one of the 12 lab-engineered mutant strains were prepared. These cultures were then grown in the absence of any antibiotics for several generations. Every 24 hr, some of each culture mixture was removed and analysed (Figure 1). More culture was also diluted into fresh antibiotic-free growth media, and the entire process was repeated for a period of 15 days. Colony counting was used to calculate the number of bacterial generations during each period.

**Figure 1.**
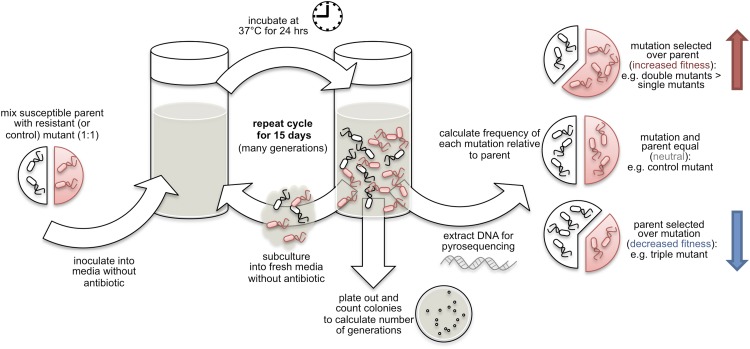
Fluoroquinolone-resistant mutants of *Salmonella* Typhi vs susceptible strains. Fluoroquinolone-resistant mutants (red) with either one, two or three mutations in the *gyr*A and *par*C genes were cultured with a fluoroquinolone-susceptible parent strain (white) in liquid media that did not contain any antibiotics. Every 24 hr, over a period of 15 days, these growth cultures were subcultured into fresh antibiotic-free media, and samples were taken for colony counting and DNA extraction. Pyrosequencing of extracted DNA was used to determine the frequency of each mutation and the parent strain at each time-point, and to calculate the fitness of each mutant compared to the parent strain. Increases in fitness allowed some single and double mutants (red arrow) outcompeted the parent strain, while a reduction in fitness meant that the triple mutant lost out to the parent strain (blue arrow).

Whether each mutant had a selective advantage or disadvantage was determined from the frequency of the engineered mutated genes at each time point, which was measured by specific pyrosequencing with the extracted DNA. Statistical models were used to calculate the selection coefficient and fitness difference of each mutant when compared to the parent strain. Since the fate of a particular mutation depends on its interaction with its environment, including all the other genes found in the genome (Cordell, 2002), the effects of interactions between genes, often known as ‘epistasis’, were also calculated for the double and triple mutants.

Interestingly, six resistant mutants (two single mutants and the four double mutants tested) could outcompete the susceptible *Salmonella* Typhi parent strain. This indicates that these mutations conferred increased fitness, even in the absence of the antimicrobial. This goes against the dogma that resistance mutations always have a fitness cost, and can explain the frequent occurrence of certain resistant mutants in the antibiotic free environment. The single mutant with highest selective advantage (called S83F) contributed 1.3% more offspring per generation than the parent strain, and the most competitive double mutant (S83F-D87N, which has two changes within the *gyr*A gene) contributed 7.4% more offspring per generation. In contrast, the triple mutant (S83F-D87G-S80I) contributed 1% less offspring per generation than the parent strain: this indicates a negative interaction between these mutations, with the third mutation wiping out the fitness advantage of the double mutants.

Since *Salmonella* Typhi lives within host cells during an infection, and is restricted to humans, the results obtained in this in vitro experimental system using a weakened, lab strain would not be the same with wild strain and ex vivo or in vivo models. However, as acknowledged by Baker et al., all models have their own inherent limitations and currently there are no available systems (except humans and primates) that exactly mimic *Salmonella* Typhi infection cycle.

The process of evolution in any organism is determined by the order in which mutations occur (Kimura, 1985). Baker et al. have concluded that single mutations in *gyr*A gene that reduce susceptibility to fluoroquinolones (especially the S83F mutation) provide the starting point for the evolution of more resistant double mutants, which maintain the resistance, even in the absence of antibiotics. This would contribute to the global spread of fluoroquinolone-resistant *Salmonella* Typhi, and would also undermine efforts to control the spread of drug-resistant typhoid through the prudent use of antimicrobial drugs.
